# Understanding Acquired Brain Injury: A Review

**DOI:** 10.3390/biomedicines10092167

**Published:** 2022-09-02

**Authors:** Liam Goldman, Ehraz Mehmood Siddiqui, Andleeb Khan, Sadaf Jahan, Muneeb U Rehman, Sidharth Mehan, Rajat Sharma, Stepan Budkin, Shashi Nandar Kumar, Ankita Sahu, Manish Kumar, Kumar Vaibhav

**Affiliations:** 1 Department of Neurosurgery, Medical College of Georgia, Augusta University, Augusta, GA 30912, USA; 2 Department of Pharmacology, Buddha Institute of Pharmacy, CL-1, Sector 7, Gorakhpur 273209, India; 3 Department of Pharmacology and Toxicology, College of Pharmacy, Jazan University, Jazan 45142, Saudi Arabia; 4 Medical Laboratories Department, College of Applied Medical Sciences, Majmaah University, Majmaah 15341, Saudi Arabia; 5 Department of Clinical Pharmacy, College of Pharmacy, King Saud University, Riyadh 11451, Saudi Arabia; 6 Neuropharmacology Division, Department of Pharmacology, ISF College of Pharmacy, Moga 142001, India; 7 Center for Undergraduate Research and Scholarship, Augusta University, Augusta, GA 30912, USA; 8 The Graduate School, Augusta University, Augusta, GA 30912, USA; 9 Environmental Toxicology Laboratory, ICMR-National Institute of Pathology, New Delhi 110029, India; 10 Tumor Biology Lab, ICMR-National Institute of Pathology, Safdarjung Hospital Campus, New Delhi 110029, India; 11 Department of Oral Biology and Diagnostic Sciences, Dental College of Georgia, Augusta University, Augusta, GA 30912, USA

**Keywords:** Acquired Brain Injury (ABI), post-birth, brain impairment, brain functions, pathologies, cellular mechanisms, therapeutic approach

## Abstract

Any type of brain injury that transpires post-birth is referred to as Acquired Brain Injury (ABI). In general, ABI does not result from congenital disorders, degenerative diseases, or by brain trauma at birth. Although the human brain is protected from the external world by layers of tissues and bone, floating in nutrient-rich cerebrospinal fluid (CSF); it remains susceptible to harm and impairment. Brain damage resulting from ABI leads to changes in the normal neuronal tissue activity and/or structure in one or multiple areas of the brain, which can often affect normal brain functions. Impairment sustained from an ABI can last anywhere from days to a lifetime depending on the severity of the injury; however, many patients face trouble integrating themselves back into the community due to possible psychological and physiological outcomes. In this review, we discuss ABI pathologies, their types, and cellular mechanisms and summarize the therapeutic approaches for a better understanding of the subject and to create awareness among the public.

## 1. Introduction 

**Figure 1:**
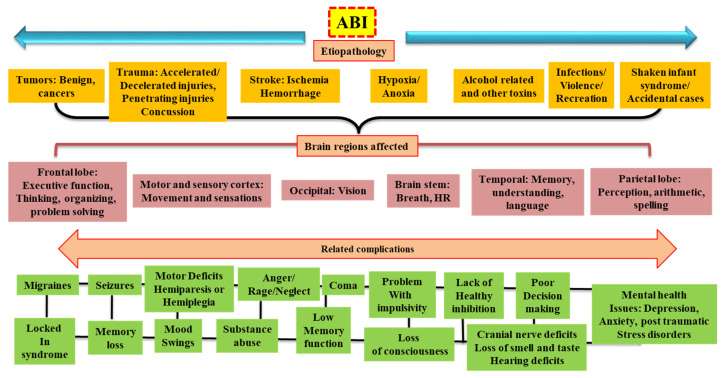
**ABI: Etiopathology, classifications, the brain region affected, and related complications.** The pictorial presentation of ABI describes its type (purple) and etiology of the disease (in orange texts). ABI is mainly divided into TBI and Non-TBI injuries. Non-TBI can arise from tumors, vessel occlusion, infection, or alcohol consumption. ABI can affect different regions of the brain depending on impact, insult, infection, or blockage (shown in pink) and may show related signs and symptoms (depicted in green). HR: Heart rate.

Acquired Brain Injury (ABI) is an umbrella term encapsulating its two main categories: Traumatic Brain Injury (TBI) or Non-Traumatic Brain Injury (Non-TBI) [1]. TBI is an external traumatic event in which injury to the brain is sustained, while Non-TBI occurs due to an internal disease process that also leads to damaged brain tissue. Causes of TBI include motor vehicle accidents, falls, sports-related injury, and violence, whereas Non-TBI could be triggered by a stroke, neoplasm, infection, and anoxia [1]. Clinical outcomes of both categories of ABI vary, depending on the specific disease process and the premorbid circumstances such as age, genetics, and socioeconomic background. Risk rates for TBI are the greatest in the elderly at and above 75 years, and male individuals are at greater odds of getting TBI [2,3,4,5]. The vectors of brain damage in both TBI and Non-TBI include vascular abnormalities, broad axonal injury, focal or disseminated atrophy, and neuronal circuit disruption [6] (Figure 1).

ABI describes a wide range of diseases, establishing it as a vastly important area in medicine and public health. According to the Centre for Disease Control and Prevention (CDC), TBI is one of the major groups of ABI and is a principal cause of mortality and lifelong disability [7,8]. As per CDC reports (2006–2014), the frequency of TBI-related hospitalizations, emergency department visits, and deaths had increased by 53 percent [9]. In 2013, roughly 2.8 million TBI cases occurred in the United States of America. Among the 2.5 million emergency department visits, there were approximately 300,000 TBI hospitalizations and about 60,000 deaths [2,10]. It is important to keep in mind that these numbers only refer to one-half of the diseases associated with ABI. Non-TBI also plays a large role in the number of individuals ending up in the hospital. The CDC reports that every year, approximately 800,000 people will have a stroke and, in 2018, one in every six cardiovascular-disease-related deaths was due to stroke [11,12]. These epidemiological data on ABIs elucidate the necessary interventions that hospitals and researchers need to accomplish to serve the large extent of individuals affected by ABI.

**Figure 2:**
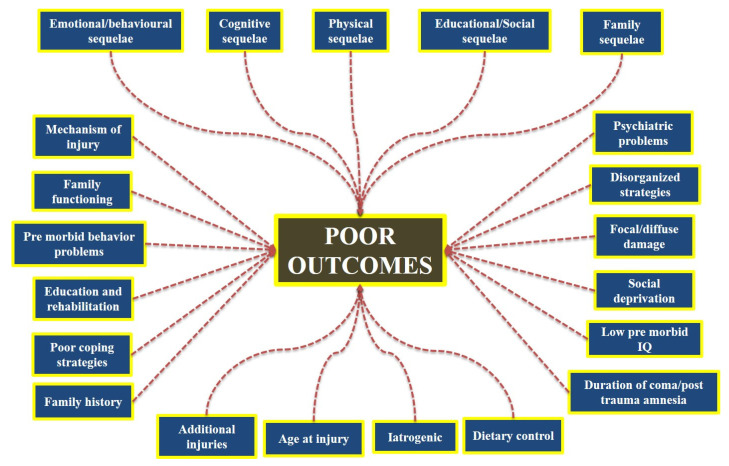
**Poor outcomes post-ABI.** A variety of parameters related to the ABI mechanism play a role in predicting the outcome. ABI can develop as a result of a stroke or disease or an iatrogenic cause, and there is some indication that those who suffer from a head injury are a self-selecting group, with poor attention, impulsivity, and overactivity being associated with poor road-crossing skills. These may interact with other premorbid characteristics that are predictors of poor post-injury prognosis.

While both TBI and Non-TBI carry many different disease processes and medical problems (Figure 2), the patients usually receive treatment and rehabilitation in the same facilities in the hospital. This is important to mention because demographic characteristics of TBI and Non-TBI vary considerably. For example, the Toronto Rehabilitation Institute demonstrated that the patient population for TBI compared to Non-TBI were significantly younger, tended to be male, and lived in metropolitan areas [13]. In addition, the global population is aging, so leaders in the medical profession need to anticipate larger demand for units and specially trained staff to treat patients with TBI and Non-Traumatic Brain Injuries with possible comorbidities [14,15]. Nevertheless, to ensure exceptional clinical outcomes for patients with ABI, physicians, and nurses must be able to provide personalized and specific treatments to the patients. To achieve that, a good understanding of ABI and its pathology in different categories is preferred. Therefore, the present review aims to give a comprehensive and clear description of ABI, its types, mechanism, and treatment strategy.

## 2. Acquired Brain Injury and Its Types

As mentioned above, ABI is a broad classifying term encompassing any non-congenital brain injury; therefore, ABI is inherently diverse in the populations it affects, in the mechanisms by which brain injury ensues, and in prognosis. The following paragraphs break down the different types of TBI and Non-Traumatic Brain Injuries that make up ABIs (Figure 1).

### 2.1. Traumatic Brain Injuries (TBIs)

TBI arises because of a hit or jolt to the brain and comprises mild to severe injury. TBI patients show symptoms such as unconsciousness, confusion, nausea, dizziness, headache, or incoordination and receive symptomatic and stabilizing treatment. Patients keep visiting clinics with chronic symptoms even after weeks or months post-initial traumatic experiences. If either symptoms continue or neurologic impairments appear, routine radiological re-imaging may be required to assess the situation [16]. There is a comprehensive review published by our group that might be of interest to the TBI audience to obtain more insight [17].

#### 2.1.1. Concussion

A concussion is one of the most widely recognized forms of TBI. It occurs due to a sudden strike or whip to the head that causes the brain to bounce or twist within the skull. Symptoms can range from minor confusion and disorientation to complete amnesia, nausea, vomiting, and loss of consciousness [18]. These symptoms occur due to abnormal brain movement upon impact, which at the molecular level disrupts neuronal cell membranes and axonal stretching. This, in turn, causes the extensive flux of ions across neuronal membranes resulting in diffuse waves of depolarization, which precipitate the classic concussion symptoms [18]. In 2006, a study on concussion epidemiology in Canada noted that 110 individuals per 100,000 had had a concussion within the previous year [19]. In 2014, 2.87 million cases of TBI in the United States were recorded by the CDC, and of those, 812,000 cases were children diagnosed with concussion alone or in combination with other injuries [2]. Similarly, according to a US study carried out in 2017, approximately 19.5% of adolescents (in grades 8–12) reported a minimum of one concussion, while 5.5% had more than one concussion in their lives [20].

#### 2.1.2. Skull Fractures

A skull fracture results from any impact to the head that surpasses the bone’s capability to withstand the pressure. Although a fracture of the skull itself is not a brain injury, over 75% of all skull fractures are associated with some form of brain injuries, such as intracranial hemorrhages or subdural or epidural hematomas [21]. Fractures that occur at the basilar skull are more problematic as this area of the skull harbors essential areas of the brain that allow us to eat, breathe, and walk [22].

#### 2.1.3. Epidural or Subdural Hematomas and Subarachnoid Hemorrhage

In general, a hematoma is a bruise, and a hemorrhage is a bleeding blood vessel. In the case of TBI, it is called an epidural or subdural hematoma if the insult occurs above or below the dura matter. Hematomas mostly occur from blunt force trauma to the head and are typically found in the temporal brain; however, they may also occur from a penetrating TBI or spontaneously (the spontaneous type would not be considered TBI) [23]. A subarachnoid hemorrhage is divided into two groups: aneurysmal vs. non-aneurysmal. The non-aneurysmal hemorrhage most often occurs due to blunt force trauma to the brain or sudden acceleration changes. Epidural hematomas account for roughly 2% of injuries to the head and 5–15% of fatal head injuries. Subdural hematomas are more common, with an estimated rate of 5–25% in patients with head trauma [21].

#### 2.1.4. Penetrating Brain Injury 

Penetrating brain injuries can be caused by assaults, collisions, or even suicide attempts [24] and may be defined from mild to severe TBI based on the Glasgow coma scale (GCS). Following a patient’s preliminary examination, neurosurgical examination starts with a clinical exam and documentation of raised intracranial pressure (ICP). In the case of suspected arterial or venous injury, a CT scan is the first choice for diagnosis. As the possibility of post-TBI epilepsy is 45–53%, a prophylactic anticonvulsant is given to patients [25].

### 2.2. Non-Traumatic Brain Injuries (Non-TBI)

#### 2.2.1. Infections

Due to the multiple layers of protection from the skull, meninges, and the blood-brain barrier (BBB), the brain is relatively repellent to any pathogenic invaders [26,27]. However, when bacteria and other pathogens breach the brain’s defenses, the damage can be devastating. Two major types of brain infections are meningitis and encephalitis. Meningitis occurs when a bacterial agent infects the meninges and encephalitis is the infection of the brain tissue itself [28,29]. Approximately 1.2 million cases of meningitis occur globally each year [30], while the incidence of encephalitis infection tends to vary between studies, but the 2019 census estimated 1.4 million cases with 89,900 deaths and 4.80 million DALYs [31].

#### 2.2.2. Anoxia

The brain needs a lot of oxygen and energy in the form of glucose. Anoxic brain injury results when the brain is completely denied of oxygen in incidences such as drowning, heart attack, carbon monoxide poisoning, and much more. As a result, the metabolic homeostasis of the brain is destroyed resulting in major neuronal injury and cell death. Since there are many different causes of anoxic brain injury, rates are hard to gauge [32].

#### 2.2.3. Stroke

There are two main types of stroke: ischemic and hemorrhagic. Ischemic strokes result from occluded cerebral arteries, which prevent nutrient-rich blood from supplying the surrounding brain tissue. This results in permanent tissue damage. Transient ischemic attack (TIA), also referred to as a mini ischemic stroke, only lasts for a short amount of time. Ischemia that affects more than two-thirds of the middle cerebral artery (MCA) territory is termed malignant cerebral infarction (MCI) and causes space-occupying edema and neurological deterioration [33]. Swelling and symptomatology peak in the first 48 h after a stroke. The first step in treatment is to reduce risk factors and keep ICP under control. Although there are no precise surgical recommendations, a hemicraniectomy is generally recommended [34]. Hemorrhagic strokes result from cerebral artery leakage into the brain, causing elevated ICP and cellular damage [35]. The incidence of stroke among adults aged between 35 to 44 years is roughly 30 to 120 per 100,000 per year. This number increases drastically for individuals aged between 65 to 74 years, where the yearly incidence is about 670–970 per 100,000 [36]. A detailed account of brain hemorrhage for further reading can be found here [37].

#### 2.2.4. Alcohol and Drug Use

The usage and abuse of alcohol and drugs are highly prevalent in modern-day lifestyles estimating a high lifetime risk for either drug or alcohol abuse and dependence [38]. There are many mechanisms through which drugs and alcohol can have negative effects on the normal functioning of the brain. These include disturbing nutrient distribution to brain tissue, direct cellular damage, altered chemical homeostasis of the brain, and hypoxia [38,39].

#### 2.2.5. Neoplasm

In a similar way to anoxia and infectious Non-TBI, brain cancers (neoplasm) are vastly diverse in pathophysiology and epidemiology. Gliomas are the most prevalent class of brain neoplasm, accounting for roughly 78% to 80% of all malignant brain tumors. These cancers stem from the supporting neuronal cells of the brain called glia. Gliomas include astrocytomas, ependymomas, glioblastoma multiforme, medulloblastomas, and oligodendrogliomas [40].

Meningiomas are the most prevalent primary tumors and are also classified as ABIs [41]. Patients with genetic predispositions to disorders, such as neurofibromatosis type 2 or multiple endocrine neoplasia type 1, are more likely to develop meningioma [42]. The preponderance is asymptomatic and histologically benign [43]. Initially, generalized symptoms (nausea, headache, or altered mental status) may be present, with localized neurological abnormalities developing later [44]. In situations with subtotal meningioma extraction, adjuvant therapy in combination with postoperative radiation may be recommended. Patients with meningioma have a good prognosis, albeit those with a higher WHO grade or partial resection have a higher chance of continuation [45].

## 3. Mechanism of ABI

A sort of physical trauma from an external entity may lead to a brain injury. The medical field has acquired tremendous success in the treatment of head injuries over the last few decades. A clearer understanding exists of the causes of tissue damage and the biophysical, biochemical, or physiological repercussions that culminate in a variety of clinical manifestations such as scalp laceration, syncope, and progression to a persistent vegetative state [46,47,48,49,50,51]. Various sorts of pathologies, such as skull fracture, hematoma (intracerebral, epidural, subdural, or intraventricular), as well as different types of contusion and brain injuries, could be recognized and their clinical and functional repercussions could be defined by contemplating the mechanisms of injury to the head [50].

### 3.1. Biophysical Mechanism of ABI

The physical characteristics of the intruding substance, such as density, size, speed, and length of loading, determine how much energy is delivered to the cranium in ABIs [52]. When a harmful energy burden or mechanical response is exerted to the head, the length of the energy loading will be the first determinant to determine the severity of the injury [53]. This period has been set between 50 and 200 milliseconds. Static loads are defined as those lasting more than 200 milliseconds, while dynamic loads are defined as those lasting less than 200 milliseconds [54,55]. Static injuries are extremely rare and mainly occur when the head is ensnared between two hard objects. These enormous weights may cause distortion and injury to the skin and bones. The dynamic load could be caused by the passage of energy to cerebral tissue via impetuous loads, which are variable in speed. When the head does not receive direct impact but is put into motion as a result of an impulse generated by a force exerted on different parts of the body, this is known as impulsive loading [56]. In such cases, the injury is caused by inertial shifts in the head. In the next category, called impact loads, when an infringing object strikes the head, it may cause tissue injury in-depth, depending on the surface area, density, size, and speed of the object. It has the potential to alter the head’s pace and induce acceleration or deceleration and may cause inertial shifts in the head too.

The influence of an object on the head can cause changes in the tissue’s arrangement, including the skin, bone, and deep frameworks. If the adjustment is greater than the tissue’s elasticity, it will lead to permanent disfigurement, skin laceration, or a bone fracture. With higher weights, the aggravating agent may produce depressed skull fractures and destruction of underlying tissue, such as the dura, brain, and arteries. This often results in an epidural hematoma, subdural hematoma, contusion, or intracerebral hemorrhage. Perforation and permeation may occur in more extreme situations, specifically at fast speeds, and with small agent sizes. The transmission of energy to the skull and cerebral tissue may be related to the collision. The brain tissues distort, and contusion could be because of this energy burden [57,58].

### 3.2. Injury to the Tissues

Tissue distortion is caused by deformation, shock waves, and acceleration/deceleration, which all impose energy on the tissue. These can cause damage to the tissues in the skull, which include neural components, vessels, and bone. Injuries arise when the stress applied to the tissue exceeds the threshold. The physical properties of tissues, the amount of energy, the length of energy loading, and the magnitude of the load all influence their endurance. More intense activities, while still within the continuum, are at the brink of physiologic endurance and, if repeated, will cause progressive or even acute brain malfunction. In normal physiological conditions, the tissues’ physical tolerance to injury is substantially lower, resulting in a variety of outcomes depending on the implicated components of pathological injury [59].

#### 3.2.1. Primary and Secondary Injuries

**Figure 3:**
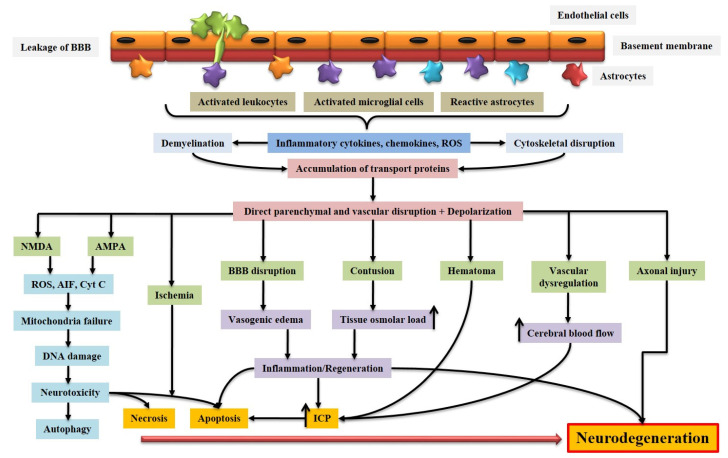
**Schematic representation of pathophysiology of ABI.** BBB dysfunction caused by injury allows the transmigration of activated leukocytes into the injured brain parenchyma, which is facilitated by the upregulation of cell adhesion molecules. Activated leukocytes, microglia, and astrocytes produce ROS and inflammatory molecules such as cytokines and chemokines that contribute to demyelination and disruption of the axonal cytoskeleton, leading to axonal swelling and accumulation of transport proteins at the terminals. On the other hand, excessive accumulation of glutamate and aspartate neurotransmitters in the synaptic space due to spillage from severed neurons activates NMDA and AMDA receptors located on post-synaptic membranes, which allow the production of ROS. As a result of mitochondrial dysfunction, molecules such as apoptosis-inducing factor (AIF) and cytochrome c are released into the cytosol. These cellular and molecular events including the interaction of Fas with its ligand Fas ligand (FasL) ultimately lead to caspase-dependent and -independent neuronal cell death. BBB: blood-brain barrier; NMDA: N-methyl-D-aspartate receptor; AMPA: α-amino-3-hydroxy-5-methyl-4-isoxazole propionic acid receptor; ROS: Reactive oxygen species; Cyt c: Cytochrome c; ICP: Intracranial pressure; AIF: Apoptosis-inducing factor.

The mechanism that causes the initial injury is a direct outcome of the delivered energy to the head [17,60]. They may cause additional injuries as a result of themselves, either as sequelae of the original event or by exacerbating it, resulting in secondary injuries, the most prevalent of which are hypotension and hypoxia. Secondary injury can include excitotoxicity, free radical formation, mitochondrial dysfunction, induction of damaging intracellular enzymes, and other pathways inside the wounded neural tissues, all of which can cause continued system dysfunction (Figure 3) [17,61,62,63]. Certain tertiary damages are also included, which are frequently secondary results of the head’s energy loading. This includes electrolyte imbalance due to renal issues, various types of cardiac abnormalities, hepatic insufficiency, and so on.

Various types of clinical cases can be distinguished based on the above-mentioned aspects in the formation of a head injury. It can begin with a bone injury, since prolonged static loading causes a change in the normal structure of the skull, ultimately leading to a fracture when the flexibility of the bone toleration is exceeded. The amount of force and the timing of the fracture determine the severity of the crack. When a large load is applied, the entire skull is severed into segments, and the brain tissue is ruptured, causing it to leak from the punctured nose, ear canals, and scalp. The sufferer may present with severe impairment of cerebral and brain stem function. Death is often the result [64,65,66].

If the injury occurs in an acoustic region, neurological deficits may occur as a result of damaged brain function. These could be the injury’s direct or major consequences. There are certain further occurrences of the above-mentioned events, which are referred to as secondary traumatic effects. Various types of intracranial hematomas, as well as intraventricular hematomas and brain tissue contusions, can occur from injury to the cerebrovasculature in the affected areas. These particulate lesions can cause a mass effect, as well as an upsurge in ICP and brain herniation [17,65,67,68]. As a primary injury, brain laceration may predispose the sufferer to convulsions and epilepsy [69,70].

Infections of the bone and cerebral contents are another serious result of this type of injury, as the overlaying skin is punctured, allowing bacteria to enter the deeper structures. These later occurrences are also secondary impacts. Lesions can occur as a result of the expansion of a hematoma or contusion, or as a result of the mass effect caused by various subsequent effects of damage, such as edema around the lesions. Cerebral herniation, concussions, diffuse axonal damage, subdural hemorrhage, intracerebral hemorrhage, and intraventricular hemorrhages are some of the other problems [71,72,73]. Severe injuries can range from a short period of disorientation and cognitive impairment to concussion or loss of consciousness to a long-term coma or persistent vegetative state (PVS) due to extensive harm to brain neurons and axons [17,74].

#### 3.2.2. Prenatal and Birth Damage

Early prenatal injury can result in the embryo’s mortality. On other hand, insufficient growth (agenesis of the corpus callosum or anencephaly) or abnormal development (lissencephaly or microcephaly) could be the possible outcomes of late injuries [75,76,77,78]. During a period of heightened development, trauma (such as fetal stroke in the womb or damage to the mother) will result in more structural issues than when progression has slowed or has ended. Intricate stent delivery or hypoxia may result in birth failure [79,80,81,82].

#### 3.2.3. Post-natal Injury

Pediatric patients may have acquired ABI from metabolic disturbances (phenylketonuria), systemic illness (sickle cell disease, diabetes), trauma, central nervous system tumors, infections (meningitis or encephalitis), toxins (use of alcohol or anticonvulsants during pregnancy), and clinical treatment such as radiotherapy or chemotherapy for leukemia [83,84,85,86,87,88,89].

#### 3.2.4. Injuries in Adulthood

According to recent CDC data, people aged 75 years or older had the highest rate of hospitalizations (325 of total TBI-related hospitalizations) and mortality (28% of TBI-related deaths) [90,91]. The CDC further stated that males are two times more likely to get TBI-related hospitalization and have three times higher mortality than females [90,91]. Alcohol intake is identified as a major risk factor for TBIs, with effects on assessment, intensity, and prognosis. It was discovered that 16 percent of brain injury patients aged 15 and above were intoxicated at the time of injury. The alcohol group had a fatality rate of 14.5 percent compared to 9 percent in the non-alcoholic group [92]. Individuals with an older ABI have a higher chance of poorer physical, intellectual, and psychosocial outcomes, as well as a lengthier recovery period and more comorbidities. The major cause of brain injuries that comes under this category is fall. More than half of all fatal falls and 8% of nonfatal fall-related hospitalizations were caused by these brain injuries. ABIs have the highest incidence of death and hospital admittance among fall-related injuries in adults and older adults during the first year after the injury. Furthermore, even after controlling for age and gender, there are rising tendencies in the incidence and mortality of trauma-induced ABIs in older persons. According to several studies, those who take anti-arrhythmic medications are more prone to suffer from brain damage. Several studies show that men had a higher chance of serious brain injuries during a fall than women, despite the possibility of a reverse relationship with nonfatal brain injuries [92,93].

The number of elderly persons hospitalized for a fracture has decreased over the last decade, whereas the percentage of those with a TBI, subarachnoid hemorrhage, and/or, subdural, in particular, has risen dramatically [94]. TBIs are becoming more common, which appears to be linked to the increased use of anticoagulants and antiplatelet medicines like clopidogrel and warfarin. Chronic illnesses related to equilibrium disturbance (Stroke and Parkinson’s disease), scenarios of falls likely to result in an ABI, and risky behaviors may happen more frequently in men, in contrast to the use of anticoagulants and antiplatelet drugs. There is a need for more investigation into the underlying principles [95,96]. It is reasonable to assume that elderly adults with chronic diseases that affect the joints, nervous system, cardiac system, and cognition are at a higher risk of falling and developing ABIs. These may also be exacerbated by a lack of visual perception and visuo-motor reflexes [97,98].

### 3.3. Physiological Mechanisms of ABI 

There are different mechanisms that arise from primary and secondary injury and contribute to ABI pathology (Figure 3). We briefly described the pathological events here to understand the pathology of ABI.

#### 3.3.1. Excitotoxicity

Glutamate, an excitatory amino acid neurotransmitter is primarily responsible for triggering cellular damage during brain ischemia. It has a multifaceted role in synaptic plasticity, brain development and maturation, axon guidance, and general neuronal growth [17,99]. In ABI, restricted blood flow to the brain diminishes energy reserves and causes membrane depolarization, thus leading to the reduced uptake of glutamate from the surroundings. Under stable conditions, glutamine activates multiple receptors such as N-methyl-D-aspartic acid (NMDA), kainic acid receptors, and alpha-amino-3-hydroxy-5-methylisoxazole-4-propionate (AMPA), while its clearance is managed by active ATP-dependent transporters [100,101]. During ABI, glutamine triggers the activation of sodium channels (causes brain swelling), calcium channels (causes neuronal death), and intracellular catabolic enzyme activity via glutamate receptors thus leading to cell death, which further cascades into the generation of oxygen free radicals, membrane depolarization, and intracellular toxicity leading to brain injuries [102,103]. Preclinical studies suggest a protective effect of suppressing NMDA and AMPA receptors post-ABI but have undesirable side effects [104,105]. To overcome this, Memantine (partial NMDA antagonist) was tested, along with death-associated protein kinase and calcium-calmodulin-dependent protein kinase, and showed potential therapeutic efficacy without many side effects [100,106]. Another dopaminergic agonist, Amantadine was found to be promising in several brain injuries. It triggers the dopamine release in neurons and delays the reuptake of dopamine by neural cells and also inhibits the NMDA receptor signaling, thus proving its potential effect in the brain injuries [107,108,109,110]. A non-psychotropic cannabinoid (Dexanabinol), which acted as a potent NMDA receptor antagonist was also reported to have a potential effect in glutamate injury, but also showed unwanted side effects that impaired normal brain functioning [37,111]. Additionally, metabotropic glutamate receptors (mGluRs) were also reported to express a promising response in retarding excitability thus hindering excitotoxicity [112].

#### 3.3.2. Oxidative Stress

A possible precursor to the pathogenesis of cerebral injury has been identified as oxidative stress. Several reactive oxygen species (ROS), such as superoxide, hydrogen peroxide, hydroxyl radicals, and per hydroxyls can be generated, followed by the development of several reactive nitrogen species (RNS), which can cause brain tissue damage through a variety of cellular and molecular pathways [17,37,102]. The reaction of nitric oxide along with superoxide forms peroxynitrite, which can also bind to DNA directly, altering its structural integrity and causing cell damage as well as apoptosis [17,113]. These highly reactive radicals can degrade nucleic acids, proteins, and lipids, leading to neuronal cell death. Edaravone and NXY-059, two promising antioxidants, were used to treat stroke but did not produce significant effects [102,114,115]. Polyethylene glycol (PEG)-conjugated SOD (PEG-SOD or pegorgotein) was reported to have promising effects in several studies but failed in a larger phase III clinical trial [17]. Another study with lecithinized superoxide dismutase (PC-SOD) showed that it inhibited secondary neuronal loss after brain injury and enhanced survival rates [116]. As a result, novel therapeutic strategies for minimizing the devastation caused by ROS are desired. Incipient interventions, such as modulation of transient receptor potential melastatin-2 channels or poly (ADP ribose) polymerase-1 control endogenous facilitators of oxidative stress [117,118]. More investigation into the neurological effects of oxidative stress could lead to new targeted therapies for the reduction of several brain injuries, especially ABIs [118].

#### 3.3.3. Acidosis

When mitochondrial respiration is disrupted, acidosis may arise as a result of lactate buildup in the cells. Acid-sensing ion channels (AICs) are activated by protons and serve as pH sensors in the body. They are amiloride-sensitive cation ports that relate to the epithelial sodium group and enable calcium and sodium to enter neurons [37,119]. About six AIC domains have been reported, with AIC1a, AIC2a, and AIC2b being expressed in the brain and spinal cord. AIC1a and AIC2s are present in high-synaptic-density areas of the brain to help with excitatory signaling and are involved in several brain injuries [119]. With their activation, neuronal cell death occurs through sodium, zinc, and calcium influx into the cell. In experimental stroke models, the inhibition of AIC1a has a longer therapeutic window, which was much more effective than currently available drug therapies [100].

#### 3.3.4. Inflammation

Inflammation may sometimes lead to brain injuries of several types including ABI [120]. Conversely, the pathogenesis of ABIs is further complicated by inflammation [121,122]. During brain injuries, there is an intense and long-lasting inflammatory response that includes the activation of microglia, development of pro-inflammatory mediators, and penetration of different kinds of immune cells into the brain tissue [17,123]. Cytokines such as interleukin IL-6, IL-1β, tumor necrosis factor-alpha (TNFα), transforming growth factor beta (TGFβ), and chemokines such as monocyte chemoattractant protein-1 (MCP-1) and cytokine-induced neutrophil chemoattractant play an important role in the pathogenesis of inflammation in neuronal cells. Depending on the type of inflammatory response and when it happens, the immune response in the brain might have a variety of outcomes [17,102]. While chronic inflammatory activities may contribute to secondary ABIs and more prolonged detrimental events, inflammation early point may be beneficial. However, elucidating the exact mechanisms of inflammatory responses is challenging, as it is a diverse set of perceptions involving inflammatory cellular components, all of which may be harmful or beneficial [124]. Broad-spectrum blockers of inflammation (AT1 receptor blockers, PPAR gamma blockers, beta-blockers, etc.), not shockingly, minimize neuronal cell damage [125,126,127]. The lack of systematic implementation progress highlights the need for a deeper knowledge of the numerous molecular and cellular pathways after inflammation. In addition, a better understanding of the different structural profiles of diverse inflammatory mediators is needed.

#### 3.3.5. Tauopathies

**Figure 4:**
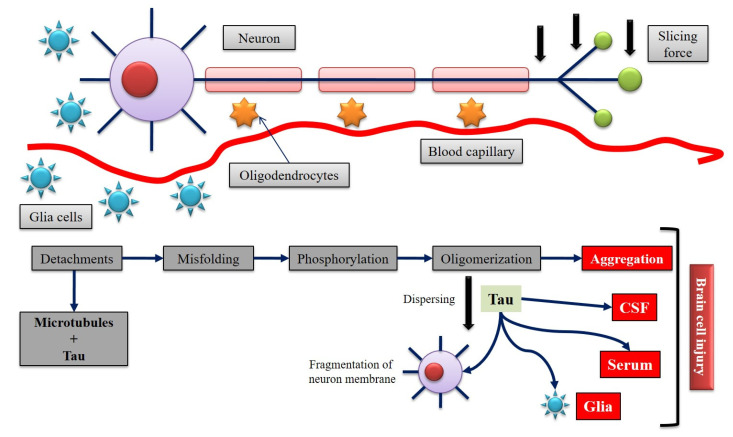
**Expected molecular mechanism of brain injury on tau in the nervous system.** Neurons, glia, oligodendrocytes, and blood vessels are damaged by the impact load that arises after a head injury. Injury to some or more of these cells causes intracellular unfolding, which causes the entire device to malfunction. Tau, which is highly correlated with microtubules, is abundant in axons. Impact forces devastate cell membrane integrity, as well as the microtubule framework in the axon. Tau disengages from the microtubule as it becomes unstable. Tau would then be misfolded, phosphorylated, develop a porous oligomeric conformation, accumulate, or disperse in a dysfunctional pathway. Tau may also invade other neighboring cells (glia, serum, or CSF) as it spreads.

Abnormal aggregation of tau proteins inside brain cells leads to several disorders including ABI [128,129,130]. The concentrations of a particular tau protein in brain tissue, CSF, and serum change in ABI pathogenesis (Figure 4) [131,132]. The events that lead to tau release can be numerous and complicated, as can the types of modified tau species. Tau’s basic role is to promote microtubule flexion and saturation, which is dependent on its post-translational modifications [133,134,135]. When Tau attaches to microtubules with a poor or no phosphorylation state; microtubule flexion is hindered; phosphorylated tau has a low potential for microtubules (Figure 1) [136,137]. Alternative splicing, which results in different-sized tau isoforms, might be another significant tau modulation [138,139,140]. Tau’s capacity to disperse amongst cells is also steered by its accumulation feature [141,142]. Oligomeric tau species disperse between cells, whereas integrated insoluble tau does not [143,144]. Tau spreads because of illness, and this property may express pathophysiological conditions triggered by ABI [145,146].

Due to the extreme sudden TBI-induced protein abundance, protein catabolic pathways such as autophagy and proteasomal degradation may become exhausted [147,148,149]. When the plasma membrane of a compromised cell is disrupted, leftover cytoplasmic proteins such as tau which leave the cell can be absorbed by neighboring cells, confirming trophic rearrangement [149,150]. Tau can cross into the cerebrovasculature, and CSF, relying on where the weakened, tau-releasing cell is located, which further tends to contribute to brain injuries [151,152,153].

## 4. Injury and Outcome

Issues may occur as a result of an ABI in a variety of ways, either directly from the injured brain or implicitly from the response of an individual to the injury. Due to changing perceptions, family factors (pre-injury family functioning and managing), psychological background, as well as social exclusion lead to an abrupt cessation of current issues [154] (Figure 2).

The foreseen consequence is aided by a variety of factors related to the mechanism of insult. Given a severe brain injury, the duration and extent of coma with a duration of post-traumatic amnesia for less than 20–30 min and with a level of the coma of 12 or less on the Glasgow Coma Scale are common characteristics of an ABI [155]. The magnitude of the damage and the functional loss normally has a dose-response correlation. Many adults and infants with traumatic complications do better than expected, whereas those with relatively minor injury issues can encounter problems. There might be conflicts between parents and professionals. Even if the injuries are minor, it is crucial not to ignore the complaints [96]. Brain injuries can attribute to low concentration, impulsivity, and overactivity which can associate with other comorbid parameters that indicate poor post-injury performance. Learning difficulties that exist pre- and post-trauma can put many individuals of discrete ages at a heightened risk, exacerbating difficulties. The most critical thing to evaluate is the observed change in behavior or educational progression [156].

### 4.1. Physical Outcomes

Aside from apparent gross motor problems, disabilities in the brain may have a significant effect on intellectual and behavioral performance. Sensory loss, weakness, tremors, seizures, excessive sweating, intermittent vision issues, ground abnormalities, and hearing problems are also possible side effects. All of these factors affect well-being, social interactions, and self-esteem [157]. Further longitudinal studies investigating these features of ABI are needed to uncover underlying mechanisms.

### 4.2. Cognitive Outcomes

Mental manifestations are among the most obscure but recurrent issues which can lead to a variety of information-processing skills—thought, speed, as well as the ability to react to tasks—being slowed down. High levels of impulsivity and impaired judgment are normal, and they have important long-term consequences [158]. Verbal communication issues may not be apparent, but there may be impaired language abilities in word searching, interpretation, and comprehension [159]. Reading, painting, structural skills, and job performance can be challenging for patients with ABIs, as well as activities and knowing physical differences. Cognition and listening abilities are often harmed. The ability to prepare, specify objectives, coordinate, and implement a plan to achieve an intended goal are examples of executive skills. This also includes expertise in efficiency surveillance and planning. Any of these issues can affect individuals or some combination of them. The severity of these functional disabilities is determined not only by the extent of the injury but also by the age at the time of concussion [160,161].

### 4.3. Educational Outcomes

Given the neuropsychological impact, the majority of rehabilitation emerges in the starting years, but developmental problems continue and can worsen. After the injury, issues about a lack of improvement in learning, speaking, and reading, as well as an inability to understand intelligent concepts and conceptualization [162]. More research is in demand to explore the underlying mechanisms.

### 4.4. Emotional and Behavioral Outcomes

Unidentified cognitive episodes and a lowered self-image linked to an understanding of logical and technical difficulties can suggest behavioral and emotional issues [163]. Making rude remarks about others may be connected with impulsivity and this behavior can be incredibly embarrassing and disconcerting for other people in society [164]. Elevated anxiety, rage, utterances of violence, fatigue, and inertia are the common and normal parts of the recovery stage and can last a significant amount of time. This is marked by a lack of enthusiasm and interest in everything, as well as difficulties maintaining focus and working at a fast pace, as well as passively carrying out recommendations rather than initiating activity. Both the extent of the incident and pre-existing symptoms can be linked to oppositional defiant disorder [165,166]. This is frequently linked to the realization of a lack of skill in certain things and being able to cope with everyday life less well [161]. The assumption by patients that they will be able to catch up with things soon can cause a lot of anxiety. Patients may be conscious of actual or potential losses, regardless of the circumstances of ABIs. Serious personal trauma can be humiliating and have a significant effect on one’s self-esteem. Fear of failure might be really serious, and it could be the origin of depression and anxiety [161]. Post-traumatic stress disorder can occur even though there is no continuous recollection of head trauma [167].

## 5. Pre-Existing Medications

Nimodipine, triamcinolone, polyethylene glycol-conjugated superoxide dismutase, and mild hypothermia have all shown positive results in phase II clinical trials [162]. Excitatory amino acid inhibitors, calcium channel blockers, NMDA receptor antagonists, corticosteroids, free radical scavengers, magnesium sulfate, and growth factors have all been used in preclinical research to evaluate the therapeutic effects of drugs in various animal models [168,169]. Regretfully, none of the formulations or methods that have been examined in phase III trials have shown to be successful [167,170]. Mannitol has been shown to help reduce brain swelling after a brain injury [171]. However, its efficacy in the long-term treatment of serious TBI is unknown. Inordinate mannitol injection has been shown to be dangerous, as mannitol passes through the circulation and the brain, increasing pressure inside the skull and worsening internal brain injuries [171]. A new meta-analysis backs up earlier reports that hypothermic treatment is a good cure for brain injuries in some situations. Health professionals should continue to use vigilance when assessing hypothermia for TBI care before more data from well-conducted trials become clear [172]. After an extreme brain injury, elevated ICP is still the leading cause of disability and death. When estimated within any intracranial space, an accelerated ICP is usually characterized as 15–20 mmHg [173]. Raised ICP has been linked to increased mortality and morbidity after extreme brain injuries. A rise in brain size at the cost of one or more intracranial resources is the cause of high ICP [174]. In ABI, increased ICP is caused by mass lesions, edema, and increased cerebral blood flow. Fortunately, there is no proof to substantiate the regular use of decompressive craniotomy in any brain injuries in adults with high ICP to increase survival and the standard of living [175]. A decompressive craniotomy can be a valuable choice when optimum medical care has failed to stop ICP. One randomized trial of decompressive craniotomy (DECRA) with extreme brain injury is currently underway, which could provide more information on the procedure’s effectiveness in adults [175].

## 6. Plausible Drug Therapies

### 6.1. S100B

The S100B protein is a member of a phenotypic family of low molecular weight calcium-binding S100 proteins that are primarily developed by glial cells. It also functions as a neurotrophic agent and a neuronal protection protein [176]. Excess supply of S100B by triggered glia, on the other hand, may exacerbate neuroinflammation and cause neuronal disruption. The brain and the serum S100B levels are scarcely associated, with serum concentrations largely determined by the blood-brain barrier’s consistency contrary to the amount of S100B in the brain [177]. Cerebrospinal S100B can be valuable as an indicator of consequence in adults with serious brain injury. Long-term functional restoration after ABI was shown to be aided by intraventricular S100B implementation [178,179]. Five weeks after brain injury, it significantly boosted hippocampal neurogenesis. The Morris water maze used to test spatial learning capacity on days 30–34 after injury, showed that an S100B injection improved cognitive efficiency [180,181]. S100B has not been used for the clinical care of any brain injury. S100B was used in a clinical trial called S100B as a Pre-Head CT Scan Screening Test After Mild TBI (NCT00717301) to see whether a serum can anticipate traumatic anomalies on a brain CT scan after a mild TBI. A change in serum S100B suggested whether the patient’s neurological condition had improved or deteriorated [182]. Finally, surgical therapy resulted in lower levels of S100B. Serum S100B protein represented the seriousness of the injury and aided in the prediction of outcomes after a serious brain injury [183]. S100B was also useful in determining the effectiveness of treatment following a serious TBI [184].

### 6.2. Statins

Statins, which are powerful inhibitors of cholesterol synthesis, can also help people with brain injuries [185]. Many of its effects, such as increased NO bioavailability, immunomodulatory activities, improved endothelial function, antioxidant properties, upregulation of endothelial nitric oxide synthase, suppression of inflammatory responses, and platelet actin reduction are cholesterol-independent [186]. Simvastatin treatment significantly increased Akt, cAMP response element-binding proteins (CREB) phosphorylation, and GSK-3; amplified the production of BDNF and VEGF in the dentate gyrus (DG); enhanced tissue regeneration in the DG; and improved cognitive and memory restoration [187]. In rats with traumatic brain injury, atorvastatin injection decreased cognitive brain abnormalities, enhanced neuronal survival and synaptogenesis in the glioma parameter range and the CA3 areas of the hippocampus, and promoted angiogenesis in these areas [188]. Pre-treatment of rats with lovastatin enhanced mental outcomes and decreased the severity of brain injury, with a concurrent decline in serum concentrations of TNF-α and IL-1β mRNA and protein [189]. In addition, statin therapy increased cerebral hemodynamics in mice after a severe brain injury [190]. Statins helped animals regain their spatial memory quickly after a brain injury. A double-blind controlled clinical trial was conducted on 21 patients with TBI (aged 16 to 50 years) who had Glasgow Coma Scale scores of 9 to 13 and intracranial deposits as evidenced by a computed tomography (CT) scan [191]. Despite the overwhelming usefulness of statins, their desirable healthcare quality profile, and comprehensive preclinical research showing both neurorestoration and neuroprotection, further clinical trials are needed to assess statins’ neuroprotective and neurorestorative properties after any type of brain injury [192].

### 6.3. Role of Phytochemicals in Brain Injury

Plants develop metabolic systems that generate hazardous and/or antinociceptive bioactive molecules as a result of their static existence and exposure to herbivores and other pathogens [193,194]. Among phytochemicals, sulforaphane (isothiocyanato-4-(methylsulfinyl)-butane) has been demonstrated to have neuroprotective effects in several experimental paradigms. Sulforaphane has shown to have a protective effect on the neurological disorder and reduces Aβ1-42-induced inflammation via nuclear factor erythroid-2-related factor 2 (Nrf2) signaling [195,196]. The putrid phytonutrients have many other effects, particularly neuroprotective, anti-proliferative, neurogenerative, anti-microbial, and allelopathic properties, in addition to their bitter taste [197]. The majority of phytochemicals formed by plants to contend with environmental factors are classified as alkaloids, phenolics, or terpenoids. There are a variety of functions and ecological positions shared by these three classes [198]. Other phytonutrients, on the other hand, readily penetrate the brain when consumed (or inhaled), where they have the capability of altering brain functions, as psychoactive phytochemicals like cannabinoids, psilocybin, and mescaline have shown [199]. These behavior-altering phytonutrients are extremely potent, acting at subtherapeutic levels and connecting explicitly to particular neurotransmitter receptors. Neuroactive phytonutrients found in widely eaten fruits, vegetables, and nuts, on the other hand, contain a bitter or sour taste that is usually well-tolerated [200,201]. Nephrotoxic phytochemicals are found in the body at concentrations much lower than their harmful level in the volume usually ingested, which explains their positive benefits [202]. Curiously, many phytochemicals are synthesized using cytochrome p450 (CYP450) enzymes [203]. It is worth noting that phytochemicals have emerged to stimulate all of the same cellular processes in mammalian cells as they have in plants. Signaling pathways involving Nrf2, SIRT1, and AMPK that developed in insects and other herbivores before humans in response to phytochemicals have been retained in human neurons [204,205,206]. Several of the compounds incorporated in the skins of fruits, according to new research, will boost cognitive function and safeguard against cognitive dysfunction in animal models of dementia, Alzheimer’s disease, and many other neurodegenerative diseases [207]. Image recognition ability was improved in old rats fed a blueberry-augmented diet, and administering green tea catechins to mice alleviated age-related contextual memory formation decline [208]. By optimizing the expression of the transcription factor CREB, both blueberry and green tea phytochemicals can strengthen cognitive performance [209,210]. Caffeine, the most commonly consumed psychoactive phytochemical, has been shown to improve cognitive performance by enhancing intracellular calcium and cyclic AMP levels, which stimulate kinases that phosphorylate and thus activate the cAMP-response element binding protein (CREB) [211].

### 6.4. Magnesium

Magnesium’s impact on calcium channels, NMDA receptors, and neuron membranes makes it a potential clinical weapon [212]. In animal studies, magnesium has been shown to improve conditions such as intellectual and sensorimotor control after a brain injury [213]. Furthermore, because of the lack of side effects and proportional effectiveness to corticosteroids, the magnesium sulfate approach has proved to be the most appropriate move [214]. Clinical trials with patients with mild or extreme TBI who reported to a level-1 community trauma unit and were assigned randomly one of two magnesium doses or placebo within 8 h of injury continued for 5 days in a double-blind study. Consistent magnesium infusions for 5 days given to patients within 8 h of a mild or extreme TBI displayed less significant effects [215].

### 6.5. Barbiturates

ICP is a risk factor for extreme ABI, and it is linked to a high risk of death. Barbiturates (pentobarbital and thiopental) are thought to lower ICP by preventing cerebral proliferation, which lowers cerebral physiological requirements and blood volume [174,216,217]. Barbiturates also lower blood pressure and can thus have an adverse impact on cerebral blood flow [218]. In one analysis, pentobarbital was considered less efficient than mannitol in lowering the ICP. In 25% of ABI patients, barbiturate therapy causes a drop in blood pressure. Any lowering ICP impact on cerebral blood flow would be compensated by this hypotensive effect [219]. Despite the fact that barbiturate coma is the secondary treatment for post-traumatic adjuvant ICP, and persistent hypotension is the most common side effect of it, recent studies indicate that low-dose corticosteroid therapy can be used in a fraction of patients to prevent hypotension [220]. ABI patients, who are plunged into a barbiturate coma, are more likely to experience adrenal insufficiency [221,222]. Some ABI patients who received barbiturates experienced adrenal dysfunction and needed higher concentrations of norepinephrine to manage cerebral blood flow than those who did not receive barbiturates [169,223].

### 6.6. Glutamate Receptor Antagonist

Neuronal cells, as a result of TBI, may become excitotoxic, which is when there is a buildup of the excitatory neurotransmitter, glutamate. This results in the overactivation of glutamate receptors causing brain damage to occur at multiple levels such as loss of the blood-brain barrier (BBB), neuron cell membrane integrity, cerebral edema, and cell death [224]. To quell the excitotoxic effects of glutamate, researchers have tried introducing a glutamate receptor antagonist (Dizoclipine), in rats with TBI [225] or topiramate in an epilepsy model in rodents [226]. The receptor antagonist was shown to alleviate continued brain damage in rodents; however, the drug was associated with an inadequate therapeutic window [227,228]. This research, although in its infancy, shows that glutamate receptors could be a viable target for TBI therapy.

### 6.7. Antioxidants

Another prominent process of secondary brain injury in TBI is through the presence of free radicals in the cerebral tissue. There are many complex mechanisms through which the injured brain produces free radicals; however, in TBI, the balance of oxidants and antioxidants is shifted [229]. The shifted balance toward oxidant production results in increased membranous lipid peroxidation, oxidized proteins, DNA damage, and mitochondrial respiration leading to neuronal cell death [17,37,230]. Researchers have demonstrated that melatonin and N-acetylserotonin have anti-inflammatory, antioxidant, and anti-apoptotic effects [231]. The administration of melatonin was shown to up-regulate antioxidant enzymes in rodent studies, which could provide a possible neuroprotective effect in humans [232].

### 6.8. Targeting Inflammation

Upon receiving a TBI, the immune system is already at work. Drugs such as dexamethasone, pioglitazone, indomethacin, or ibuprofen have shown to have prominent effects on ABI-induced inflammation [233,234]. Damage-associated molecular patterns (DAMPs), chemokines, and cytokines flush through the neural tissue, recruiting armies of white blood cells to help clean up the injury. While the intent of the WBCs is to save the brain from further disastrous damage, activated microglia (macrophages in the brain) release reactive oxidative species and excitatory neurotransmitters that contribute to further cell death [235]. Researchers are targeting inflammatory cytokines such as IL-1β and TNF-α. These studies show some promise as they were able to reduce neurologic damage and even improve cognition and motor ability [236]. Another avenue of research of neurologic immunology in TBI is through the endocannabinoid system, which has been shown to play an essential role in the homeostasis of the cell and may play a prominent role in cellular repair mechanisms after or during disease processes [237]. Endocannabinoid clinical trials failed to show significant protective effects; however, tests of synthetic endocannabinoid receptors showed some therapeutic promise in rodent studies [17,238].

### 6.9. Programmed Cell Death Inhibitors

Apoptosis of neuronal cells is another result of TBI and is considered a poor prognostic factor. Studies are targeting different areas of the apoptotic cascade. The design of drugs that inhibit cyclin-dependent kinases (regulators of the cell cycle) showed potential therapeutic outcomes as they slowed the progression of neuron death and improved health outcomes in TBI mice [239]. Another area of the programmed cell death pathway researchers are targeting for therapies is that of caspase-dependent apoptosis. Caspase-3 and 12 inhibitors (peptide-based inhibitors such as z-VAD-fmk, and qVD-oph and the small molecule inhibitors, IDN-6556 and p35) have improved health outcomes in a hemorrhagic and TBI model of rodents and can be used as an effective therapy due to its wide therapeutic window [17,240].

## 7. Future Prospective

Because of the breadth of diseases and pathophysiological mechanisms, ABI encompasses there is a range of different therapeutic options specified for each disease process. These treatments range from chemotherapies to surgical interventions. However, when it comes to TBI, contemporary treatment options are limited due to the innate complexity of TBI pathophysiology [241]. Due to this complicated nature of TBI care, modern-day intervention plans are generalized approaches that may be able to address the primary brain damage (occurring due to direct brain damage after immediate impact) but customarily fail to impede secondary neuronal tissue damage (damage that continues months to years after the traumatic episode) from the brain’s response to the traumatic event. The following covers the current treatments for TBI and their strengths and weaknesses, in addition to identifying promising future therapies.

### 7.1. ICP Monitoring and Management

ICP is the pressure that results from the closed system of the craniospinal compartment. Increasing pressure can produce disastrous amounts of stress on the brain tissue causing neuronal damage and possible brain herniation. Within the skull, there is a precious balance in secretion, composition, and volume of CSF [242]. Increased ICP is a pervasive result of TBI and is a significant result of secondary brain injury; therefore, monitoring and managing its levels is established as a critical aspect of TBI treatment. There are multiple methods healthcare providers use to monitor ICP. Computed tomography (CT) scans are often used to visualize the increase in pressure while intraventricular catheters are the “gold standard” for ICP monitoring. The catheter is usually surgically placed into the lateral ventricle where a standard pressure transducer will monitor pressure changes [243]. The catheter can also double as a drain, which can be used for the therapeutic draining of the intracranial space or, as mentioned above, for diagnostic objectives [244]. In addition to catheter use as a means of ICP therapy, head elevation is used to displace much of the CSF from the cranium and encourage venous return to the heart. With head elevation, ICP may be reduced without the disruption of cerebral blood flow [245]. Hyperventilation is another means through which ICP can be reduced medically. Hyperventilation lowers the ICP by increasing the intraarterial CO_2_ partial pressure which signals the sympathetic nervous system to vasoconstriction; however, it is only used for brief periods, when the brain tissue is under stress [246].

### 7.2. Medically Induced Coma

The brain is the single largest organ in the form of glucose consumer in the body. Accounting for roughly 2% of the body’s weight, it nonetheless consumes 20% of the body’s glucose [247]. This shows how active the human brain is. In traumatic episodes, it is important to preserve its function by reducing the metabolic demand of the brain. To do this, physicians often administer benzodiazepines or infuse barbiturates to induce a coma in the patient. This can save brain tissue from excitotoxic events and seizures, saving a great amount of neuronal tissue [246,248].

### 7.3. Surgical Intervention

As mentioned above, hematomas and hemorrhages are often associated with TBI. If the significant mass effect from a hematoma or bleeding is appreciated in imaging, then surgery is warranted. A hematoma may continue to grow, which could apply large amounts of pressure to the brain tissue resulting in neuronal death. If the hematoma begins to expand rapidly this is considered a neurosurgical emergency and pressure must be relieved via decompressive craniotomy [244].

ICP monitoring and management, medically induced comas, and surgical interventions are adequate means of immediate therapy; however, their limitation is that they fail to address the secondary effects of TBI. These effects can last from months to years and can result in neuronal cell dysfunction and neurodegeneration. There are currently little to no therapies that adequately address the main pathophysiologic mechanisms of secondary brain damage; nevertheless, many researchers are working to address this aspect of TBI treatment by addressing the different aspects of disease phenomenon such as excitotoxicity, oxidative stress, inflammation, and programmed cell death [17].

### 7.4. Remote Ischemic Conditioning as an Adjuvant Therapy

Remote Ischemic Conditioning (RIC) is a non-invasive adjuvant therapy particularly useful in treating ischemic and hemorrhagic injuries [249,250]. Recent studies validated the therapeutic effect of RIC on the treatment of several brain disorders such as focal ischemia [251,252], acute ischemic stroke [253,254], aneurysmal sub-arachnoid hemorrhage [255,256,257], and intracranial arterial stenosis [258] and in the prevention of stroke-associated pneumonia [259]. It consists of repeated cycles of temporary ischemia-reperfusion in the arms or legs. The procedure involves a manual or electronic tourniquet, which applies a pressure of 30 mm of Hg above the systolic blood pressure to establish repeated cycles of occlusion and reperfusion [249,250].

The principle of RIC, as both pre-and post-conditioning, has been validated in different in vitro, preclinical, and clinical studies in distinct disease models such as myocardial, pulmonary, and endothelial injury [260,261]. Multiple mechanisms have been put forward to explain the therapeutic effect of RIC and might involve the release of humoral factors such as nitric oxide or biogenic amines such as ornithine, glycine, kynurenine, spermine, carnosine, and serotonin [262]. These factors modulate the systemic immune response by regulation neutrophils activation [263,264], macrophage polarization [265], and/or T cell activation [266]. These mediators are also transported through the bloodstream towards the site of injury, where they attenuate disease progression by regulating multiple pathways at cellular and molecular levels [267]. This involves changes in mitochondrial metabolism characterized by a reduction in the levels of glycerol, a decrease in the lactate/pyruvate ratio, and a reduction in the rate of ATP depletion through the regulation of KATP channels [268]. At the molecular levels, it regulates distinct pathways such as AMPK [269], opioid pathway [270], Notch signaling [271], and peroxisome proliferator-activated receptor (PPAR) gamma [272]. RIC also regulates gene expression at the site of injury at both genetic and epigenetic levels. It downregulates the expression of genes associated with the regulation of metabolism, molecular transport, oxidative stress, and cell cycle regulation [273]. In the case of brain injury, its clinical relevance produced results in several clinical trials and was discussed in detail in the recently published review article by Baig S et al. [249]. While the research on the effect of the RIC on ABI is still in its infancy, further progress in this area requires investigating which patient groups respond best to RIC, identifying the optimal protocol such as dose and duration of therapy, and establishing biological and radiological biomarkers of the conditioning response.

### 7.5. Elastin Derived Peptides in Acquired Brain Injury

Elastin-derived peptides (EDPs), the fragmented product of elastin protein, have been implicated in the progression of neurological degeneration during acquired brain injury. EDPs are detectable in CSF and blood in healthy people and patients after ischemic injury [274,275]. Elastin is the major structural matrix protein found on the surface of arteries, lung tissue, cartilage, elastic ligaments, brain vessels, and skin. Due to extensive crosslinking, elastin is highly insoluble and has a long half-life [276,277]. However, under normal and disease conditions, it is degraded by serine protease (also known as elastase) [278], cathepsins [279], and matrix metalloproteases (MMPs) particularly MMP-2, -3, -7, -9, -10, and -12 [280,281].

During brain injury, elastase is released from interstitial and inflammatory cells, and together with cathepsins and MMPs degrades elastin to release EDPs [276,282]. EDPs bind to the cell-surface protein complex consisting of elastin-binding protein (EBPs), cathepsin A, and neuraminidase (Neu1) [283,284]. Another EDP receptor, Galactin-3, is expressed in inflammatory cells and is associated with tumor progression and metastasis [285,286,287]. Other less characterized EDP-binding proteins are integrins αvβ3 and αvβ5 [288,289]. The immune cells recognize EDPs as a foreign antigen and produce anti-elastin antibodies that result in an autoimmune reaction as seen during psychiatric diseases and other neurodegenerative disorders such as Alzheimer’s disease [290,291,292].

The binding of EDPs to their receptors in astrocytes activates various intracellular signaling pathways such as peroxisome proliferator-activated receptor gamma (PPARγ) [293] and AHR [294]. Together, these pathways regulate cellular activities such as cytotoxicity, apoptosis, cell proliferation, and metabolism [295]. Limited studies have investigated the effect of EDPs on nervous system cells, particularly in astrocytes. An in vitro study suggested that astrocytes express EBPs, which might be involved in the process of astrocytoma invasion [296]. In the mouse cortical glial cells, EDPs peptide decreased the expression of *Mmp-2* and *Mmp-9*, whereas it increased the expression of *Timp-2*, *Timp-3*, and *Timp-4* mRNA indicating its inhibitory effect on neovascularization [297]. The EDPs also decreased NO production and increased ROS production in astrocytes [298]. Further studies suggested that EDPs reduce the proliferation of undifferentiated neuroblastoma cells, thereby promoting aging which may underlie several neurodegenerative diseases [299].

Taken together, the studies so far indicate pro-inflammatory and anti-angiogenic effects of EDPs in brain injury. How EDPs levels in different pre-clinical and clinical ABI models affect the outcome of the diseases is worth further investigation.

### 7.6. Stem Cell—Contemporary Clinical Trial

Early studies of stem cell therapies in brain injury focus on the restoration and rehabilitation of damaged neuronal connections. Recently, the paradigm of stem cell therapy has shifted from the reconstruction of neural networks to the reduction of the secondary immune response, responsible for secondary brain injury and neurodegeneration [300]. Studies showed that hematopoietic stem cells attenuated proinflammatory markers like TNF-α and IL-β. In turn, this resulted in lower immune cell infiltration of the brain and decreased activation of microglia [301]. In cellular therapy studies, researchers found that the infusion of multipotent adult progenitor cells (MAPCs) after TBI in rodents achieved some level of neuroprotection from the secondary immune response. The results showed that MAPCs led to the efflux of IL-4 and IL-10 cytokines which stimulated the M2-microglial phenotype and MAPCs led to modulated microglial activity in neural tissue [302]. Currently, the same researchers are carrying out clinical trials with the use of mononuclear cells derived from bone marrow as a treatment for TBI. In phase 1, the bone marrow mononuclear cells were delivered intravenously to pediatric patients with a severe traumatic brain injury which showed some central nervous system preservation. Phase 2 clinical trials were completed in adults with the same functional, structural, and neurocognitive outcomes measured as in phase 1 [302].

The new developments of different therapies, targeting different aspects of the complex nature of TBI are important as they will help save the lives of millions worldwide. These possible treatment options will also give physicians more options in treatment which may be personalized to the patient’s specific injury and bodily response to said damage.

## 8. Conclusions

The established medical treatment of ABI patients consists primarily of advanced prehospital treatment, comprehensive clinical care, and long-term recovery; however, there is no scientifically validated successful management of neuroprotective agents to prevent subsequent injury or improve healing. The massive impact of ABI, on the other hand, clearly demonstrates the need for certain neuroprotective and/or neurorestorative strategies or therapies.

Combining therapies can lead to improved results. Several agents and cells or even other strategies may be used in these possible permutations. Inadequacies in clinical trial designs and analyses can affect the outcome. In new clinical trials, a more responsive interpretation of the outcome is needed with substitute performance indicators and new forms of outcome analyses. Certain phytochemicals’ potential to stimulate certain beneficial stress response mechanisms that exercise and energy restriction suggests that they can enhance brain performance and reduce the risk of neurodegenerative diseases. These neurodegenerative diseases, including Alzheimer’s and Parkinson’s disease, show that oxidative stress, reduced immune bioenergetics, mitochondrial function, and the aggregation of protein aggregates all play a role in the malfunction and continued degeneration of the brain. There are mounting results showing that neurohormetic phytochemicals have the capability to stimulate mechanisms that resist or restore oxidative damage, boost bioenergetics, and improve the elimination of proteopathic proteins like amyloid peptide and synuclein, which is helpful in improving the patient’s condition. Additionally, stem cell therapy and non-invasive RIC showed promise in improving ABI. However, extensive and robust research is needed to investigate a number of significant, unanswered questions about the underlying neural mechanisms of action of particular phytochemicals, as well as their therapeutic effectiveness in animal experimental models and human beings. Further advancement of scientific proof therapies and application of these recommendations is likely to increase the likelihood of quantitatively effective agents showing promising effects in potential clinical trials.

## Data Availability

Not applicable.
